# Using machine learning to understand microgeographic determinants of the Zika vector, *Aedes aegypti*

**DOI:** 10.1371/journal.pone.0265472

**Published:** 2022-12-30

**Authors:** Jagger Alexander, André Barretto Bruno Wilke, Alejandro Mantero, Chalmers Vasquez, William Petrie, Naresh Kumar, John C. Beier

**Affiliations:** 1 University of Miami Department of Public Health, Miami, FL, United States of America; 2 Laboratory for Computational Epidemiology and Public Health, Department of Epidemiology and Biostatistics, Indiana University School of Public Health, Bloomington, IN, United States of America; 3 Miami-Dade County Mosquito Control Division, Miami, FL, United States of America; Institute for Advanced Sustainability Studies, GERMANY

## Abstract

There are limited data on why the 2016 Zika outbreak in Miami-Dade County, Florida was confined to certain neighborhoods. In this research, *Aedes aegypti*, the primary vector of Zika virus, are studied to examine neighborhood-level differences in their population dynamics and underlying processes. Weekly mosquito data were acquired from the Miami-Dade County Mosquito Control Division from 2016 to 2020 from 172 traps deployed around Miami-Dade County. Using random forest, a machine learning method, predictive models of spatiotemporal dynamics of *Ae*. *aegypti* in response to meteorological conditions and neighborhood-specific socio-demographic and physical characteristics, such as land-use and land-cover type and income level, were created. The study area was divided into two groups: areas affected by local transmission of Zika during the 2016 outbreak and unaffected areas. *Ae*. *aegypti* populations in areas affected by Zika were more strongly influenced by 14- and 21-day lagged weather conditions. In the unaffected areas, mosquito populations were more strongly influenced by land-use and day-of-collection weather conditions. There are neighborhood-scale differences in *Ae*. *aegypti* population dynamics. These differences in turn influence vector-borne disease diffusion in a region. These results have implications for vector control experts to lead neighborhood-specific vector control strategies and for epidemiologists to guide vector-borne disease risk preparations, especially for containing the spread of vector-borne disease in response to ongoing climate change.

## Introduction

The Zika virus was the culprit for a recent public health emergency, with over 80,000 reported cases in 2016 alone [1]. The disease associated with Zika virus in humans generally presents mild symptoms lasting for up to one week [2]. However, the virus is of particular risk to pregnant women, whose children have a greater risk of birth defects [3]. Though case numbers have declined since 2016, there are still questions remaining about the rapid global outbreak [4].

In June of 2016, Miami-Dade was the site of the first confirmed Zika virus disease cases in the United States [5, 6]. Unique variations were observed across Miami-Dade County in the spread of the Zika virus. Zika is a vector-borne disease, primarily spread during the bloodmeal of female *Aedes* mosquitoes. In the United States, *Aedes aegypti* is the most important mosquito species for the transmission of the Zika virus [7]. Local transmission was generally confined to certain regions across the county and was stopped after mosquito control through the implementation of aerosol insecticides [5, 6]. According to our best knowledge, there are currently no studies examining why the 2016 Zika virus outbreak in South Florida was concentrated in select areas. It is of particular significance to understand the spread of vector-borne disease in Miami-Dade County, as vector mosquito populations in Miami-Dade County may serve as an entry point for vector-borne diseases into the continental United States [8].

Variations in vector mosquito abundance and vector-borne disease spread have been modeled for decades, especially with relation to meteorologic or climatic variables [9–15]. Studies across different continents and habitat types show significant correlations between vector mosquito abundance and temperature, humidity, and precipitation. Though some mechanisms for these variations remain unresolved, some proposed mechanisms include different activity levels and reproductive rates in different temperatures and humidities, different availability of aquatic larval habitats with precipitation, and changing interspecies interactions with weather conditions [8].

Vector species abundance also varies with microgeography, or on the scale of an individual organism’s travel distance, as different habitats offer different ecology, resource availability, and larval habitat potential [16, 17]. Microgeography is especially important in urban environments as a species’ travelling range, which for *Ae*. *aegypti* is already short (on the order of 100 meters), is shortened by the presence of manmade barriers [18]. However, urban environments have unique properties that can often support vector species [19–21]. In Miami-Dade County, ongoing research has shown that features of the urban environment such as tire shops [22], cemeteries [23], urban farms [24], and ornamental bromeliads [25] all provide aquatic habitats for mosquito development [26]. A significant reason why these habitats are able to support *Ae*. *aegypti* is due to their relationship with weather and climate; water is a determining factor in the physical environment and is necessary for them to breed, while temperature inherently affects mosquito energy expenditure, activity, and physiology [27]. Thus, while the urban environment has been studied extensively as a habitat for vector mosquitoes, it is important to understand how weather patterns interact with various urban microgeographies, such as those where buildings are denser or those that offer more vegetation, to in turn influence mosquito abundance.

This better understand the relationship between vector species, urban environments, and weather patterns, *Ae*. *aegypti* populations in various regions throughout the 2016 Zika outbreak are studied here. Long-term trends are analyzed separately in *Ae*. *aegypti* populations in regions affected by Zika during the outbreak and in regions that were unaffected. It is hypothesized that different regions across Miami-Dade County, particularly those that sustained local transmission of Zika virus and those that did not, have different population trends (differences in the mean and variance of *Ae*. *aegypti* as well as differences in observed temporal patterns). It is also hypothesized that weather variables have differing relationships with *Ae*. *aegypti* populations in Zika affected regions as opposed to *Ae*. *aegypti* populations in unaffected regions.

To assess these hypotheses, a machine learning approach was used. Random forest, a supervised learning method, can be used for multivariate regression analyses and determining the relative importance of multiple predictor variables on an outcome [28]. So far, there has been limited use of this approach for modelling the relationship between vector-species population and vector-borne disease [29, 30] but there has been wider application of this technique to other aspects of vector-borne diseases [31–33]. Some extensions to the random forest method have been specifically implemented for spatial analysis [34]. This novel method may provide a better approach to understanding the complexity of *Ae*. *aegypti* population drivers in urban habitats. Understanding the patterns that control vector species’ behavior and population dynamics is critical to mosquito control efforts and beneficial to public health, especially as vector-borne diseases have the potential to spread at increased rates and over broader distributions due to climate change [35].

## Material and methods

BG-Sentinel traps (BioGents, Regensburg, Germany), baited with CO_2_ from dry ice, were utilized around Miami-Dade County to collect data on *Ae*. *aegypti* populations [36]. Around the county, 172 traps were placed in total, with 54 placed in the areas sustaining local transmission of Zika during the 2016 outbreak and 118 placed in the unaffected region, or non-Zika region. Data were collected weekly over a period from July 2016 to March 2020. Fieldwork and data collection were done by the Miami-Dade County Mosquito Control. This study did not involve any endangered or protected species and did not put any participants at harm, so the study was deemed exempt from institutional review board assessment (IRB Protocol Number: 20161212) by the Institutional Review Board at the University of Miami.

**Fig 1:**
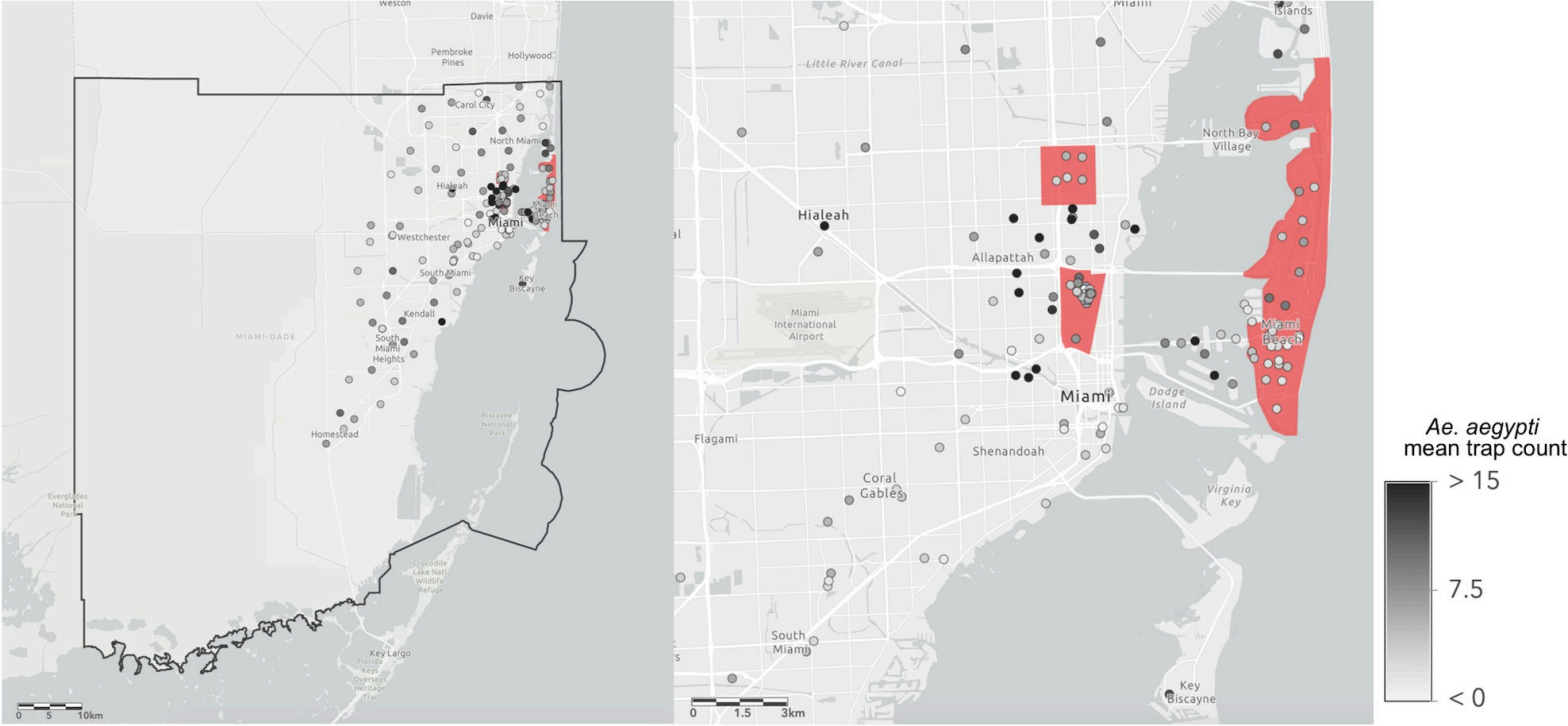
Map of Miami-Dade county mosquito control traps. The map on the left includes all 172 traps from which data was collected for this study. The black bold line represents the boundary of Miami-Dade County. The map on the right highlights areas (shaded red) that sustained local Zika virus transmission during the 2016 outbreak. Trap locations are shaded according to the mean value of *Ae*. *aegypti* collected by that trap over each collection during the study period (2016–2020).

While regular mosquito surveillance was conducted in Miami before the Zika outbreak in 2016, collection became more intensive in response to the Zika virus outbreak. Data is not compared before 2016 as traps used before the outbreak were not the same model as the BG-sentinel traps implemented after the outbreak. Variation in collection power between trap models, which some studies demonstrate, would make comparison difficult or impossible [37–40]. It is also of note that because the traps were placed in response to the Zika outbreak, mosquito traps were placed in areas affected by Zika about a month before they were placed in unaffected areas. Additionally, the start dates and end dates of each trap’s implementation varied. A full map of trap locations is included in the Results section (Fig 1). While trap locations were limited to areas where volunteers were willing to keep the trap on their properties, active traps were consistently placed at least 50 meters apart. According to the manufacturer, each trap has a maximum collection range of 20 meters, so the minimum of 50 meters distance is sufficient to prevent overlap between collections and keep sampling power constant [41]. Traps were deployed weekly for 24-hour periods. Mosquitoes were sexed and identified by species by Miami-Dade County Mosquito Control. As the traps were baited with CO_2_ to attract adult female *Ae*. *aegypti* searching for a blood meal, any male mosquitoes in the traps were considered coincidental and uninformative for surveillance purposes. They were therefore excluded in the data used for this study.

Daily high temperature, daily low temperature, and daily total precipitation data over the period from June 2016 to March 2020 were gathered from National Oceanic and Atmospheric Administration Online Weather Data, as accessed in May 2020 [42]. Daily average wind speed and relative humidity were gathered from the National Centers for Environmental Information Local Climatological Data as accessed in May 2020 [43]. Weather data is only taken from one location in Miami-Dade County for this analysis. The original collection site of each of these data was Miami International Airport, Station USW00012839, which is located at 25.7881° N, 80.3169° E.

Land-use data was accessed online from Miami-Dade County Open Data Hub [44]. Three categorical variables were used to describe land use in this study. The first was “major land use” or a description of the site where the trap was placed. This variable had 11 values: ‘Low-Density Residential (LD),’ ‘Medium-Density Residential (MD),’ ‘High-Density Residential (HD),’ ‘Business,’ ‘Education,’ ‘Religious,’ ‘Attraction,’ ‘Hospital,’ ‘Industrial,’ ‘Park,’ and ‘Vacant.’ The other two categorical land-use variables are minor land-use A and minor land-use B. To acquire the minor land-use variables, the two dominant land-use types within a 100-meter radius of the original trap site were categorized, with minor land-use A representing the highest represented land-use in the surrounding area, and minor land-use B representing the second highest represented land-use in the surrounding area. These minor land-use area categorical variables were included to show habitats around the trap site where mosquitoes might live, breed, or access other resources necessary for their survival. Data for these variables was collected using ArcGIS Pro version 10.8.1 [45]. All of the categories of the major land-use variable remain the same for minor land-use A and minor land-use B except for the addition of ‘Road,’ ‘Lake,’ ‘Rail,’ and ‘Bay,’ which are categories for either variable. In the case that all the land within 100 meters of the trap was categorized as only one type, that one type was recorded for major land-use and both minor land-uses. Per capita income data was acquired from the American Community Survey (2015–2019) [46] and is provided by the census tract. These data were accessed online through a third-party interface [47] and verified for accuracy against the original census data.

Each trap is sorted as belonging to one of two regions: areas that were affected by local transmission of Zika virus during the 2016 outbreak (“Zika region”), and areas that were not affected by local transmission of Zika virus during the 2016 outbreak (“non-Zika region”). The exact boundaries of these locations are shown in the results. Further, data are grouped into six sub-regions; three areas affected by local Zika virus transmission (Little River, Miami Beach, Wynwood), and three unaffected (Downtown/Brickell, Hialeah, Homestead). Trap counts from each region and sub-region were fitted to a Poisson distribution. Standard deviation and λ statistics for the Poisson distributions were calculated, including a 95% confidence interval for λ. The λ statistic is the mean or expected value of a poisson distribution. For this study, it represents the expected trap counts for a given region and can be used to compare observed populations between regions. This analysis was conducted using the MASS package 7.3–54 [48] in R version 4.11 [49] run through R studio version 1.4.1717 [50].

A time series plot was produced by aggregating all trap counts in the Zika and non-Zika regions to the weekly level and then creating a three-week moving average curve of the data in Microsoft Excel for Mac version 16.54 [51]. A temporal autocorrelation plot was produced for the weekly aggregated data using the stats package version 3.6.2, one of the core packages in R [49].

Note here that a “collection” throughout the remainder of the study is defined as the data acquired from one individual mosquito trap over one sampling day. A data frame was constructed by co-locating the *Ae*. *aegypti* adult female counts collected at each individual trap collection with appropriate spatial variables (major land-use, minor land-use A, minor land-use B, and per capita income), as well as temporal variables (average wind speed, daily maximum temperature, daily minimum temperature, relative humidity, precipitation) on the day of collection, one day before collection, seven days before collection, fourteen days before collection, and twenty-one days before collection. The choice of these lag times is intentional to provide weather conditions over the adult life and breeding cycle of this species. Lag times in between 0, 1, 7, 14, and 21 days are avoided as excess autocorrelation between variables would reduce the interpretability of random forest models. A table of all explanatory variables included in analyses is shown below (Table 1).

**Table 1:** Variable names, abbreviations, and sources for each explanatory variable. Each of these variables is used in random forest predictive models against *Ae*. *aegypti* trap counts. Numbers next to an abbreviation correspond to lag times used (days before collection). Further explanations of each variable are provided in methods.

Variable	Abbreviation(s)	Source	Spatial/Temporal
Average daily wind speed	AWND (0, 1, 7, 14, 21)	NCEI Local Climateological Data [43]	Temporal
Daily maximum temperature	TMAX (0, 1, 7, 14, 21)	NOAA NOW Data [42]	Temporal
Daily minimum temperature	TMIN (0, 1, 7, 14, 21)	NOAA NOW Data	Temporal
Average daily relative humidity	RH (0, 1, 7, 14, 21)	NCEI Local Climatological Data	Temporal
Total daily precipitation	PRCP (0, 1, 7, 14, 21)	NOAA NOW Data	Temporal
Major land-use	LUM	Miami-Dade County Open Data Hub [44]	Spatial
Minor land-use A	LUmA	Miami-Dade County Open Data Hub	Spatial
Minor land-use B	LUmB	Miami-Dade County Open Data Hub	Spatial
Per Capita Income	PCI	US Census Bureau–American Community Survey [46]	Spatial

Random forest analyses were performed using the RandomForestSRC package version 2.12.1 in R [52]. Three random forest models were created: one for all trap counts, a second for only those in the Zika regions, and a third for only those in the non-Zika regions. For each model, all explanatory variables listed in the data frame above were used as predictive values. Individual trap, region, sub-region, and date were not used as predictive values as the inclusion of any of these values would detract from understanding the effects of the selected weather and spatial variables on mosquito counts across each region. Variable importance (VIMP) scores shown are normalized by running two new random forest models over the same predictive variables but instead against the z-score of *Ae*. *aegypti* trap counts. These separate z-score based random forest models are only used for VIMP score calculations.

To compare the differences between the random forest models generated for Zika and non-Zika regions, cross-prediction was performed between the models. This methodology is less common because random forest models are often compared with another model type over the same data training set [31, 53]. Instead, here, two random forest models are being compared which have been trained over different data sets. To compare the random forest models for the Zika and non-Zika regions requires 1) the predictions of the Zika model 2) the predictions of the non-Zika model 3) the true trap observation data. These comparisons are performed for trap observations in both the Zika and non-Zika regions and plotted in Fig 6. Using base R, root-mean-square error (RMSE) was calculated for each set of predictions to compare model effectiveness in each region. Finally, test scenarios are presented to demonstrate the different predictive outputs of each model. A test scenario here is defined as a set of values on the models input variables (i.e. the model is presented with values for each of the weather variables and land use variables). These test scenarios are chosen to ensure they are within the appropriate sample space of the model. Specific input conditions are included as S1 Table.

## Results

**Fig 2:**
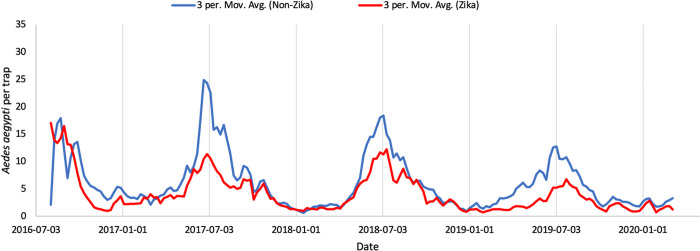
3-period moving average curve of *Ae*. *aegypti* per trap. Conducted for daily average of all traps in areas affected by local Zika transmission during the 2016 outbreak (red) and unaffected areas (blue).

In Zika and non-Zika regions, 118 and 54 traps were placed respectively (Table 2). In total, in the Zika and non-Zika regions, there were 15,213 and 11,154 collections respectively. Trap counts show that there is spatial (Fig 1) and temporal variation in *Ae*. *aegypti* across Miami-Dade County (Fig 2). The 95% confidence intervals indicate that the λ statistic for Zika regions was significantly lower than non-Zika regions (p < 0.05) (Table 2). The λ statistics were significantly different across all sub-regions except for Miami Beach and Little River (p < 0.05).

**Table 2:** Summary statistics for *Ae*. *aegypti* collected at areas across Miami-Dade County. Data are grouped into locations affected by local Zika virus transmission during the 2016 outbreak and unaffected. Areas are further subdivided into six defined regions. Trap counts from each region were fitted to a Poisson distribution. Appropriate statistics and 95% confidence intervals for λ are shown.

		Non-Zika					Zika				Total
		Downtown	Hialeah	Homestead	Other areas	Total	Miami Beach	Little River	Wynwood	Total	
**Number of traps**		8	6	5	99	118	28	5	21	54	172
**Number of collections**		1085	923	802	12403	15213	6775	1079	3300	11154	26367
***Ae*. *aegypti* per trap**	**λ**	3.02	7.82	5.76	6.63	6.40	4.00	3.95	5.00	4.29	5.51
	**Stand-ard error**	0.05	0.09	0.08	0.02	0.02	0.02	0.06	0.04	0.02	0.02
	**95% CI for λ**	(2.92, 3.12)	(7.64, 8.00)	(5.59, 5.93)	(6.58, 6.68)	(6.36, 6.44)	(3.95, 4.04)	(3.83, 4.07)	(4.93, 5.08)	(4.25, 4.33)	(5.48, 5.54)

**Fig 3:**
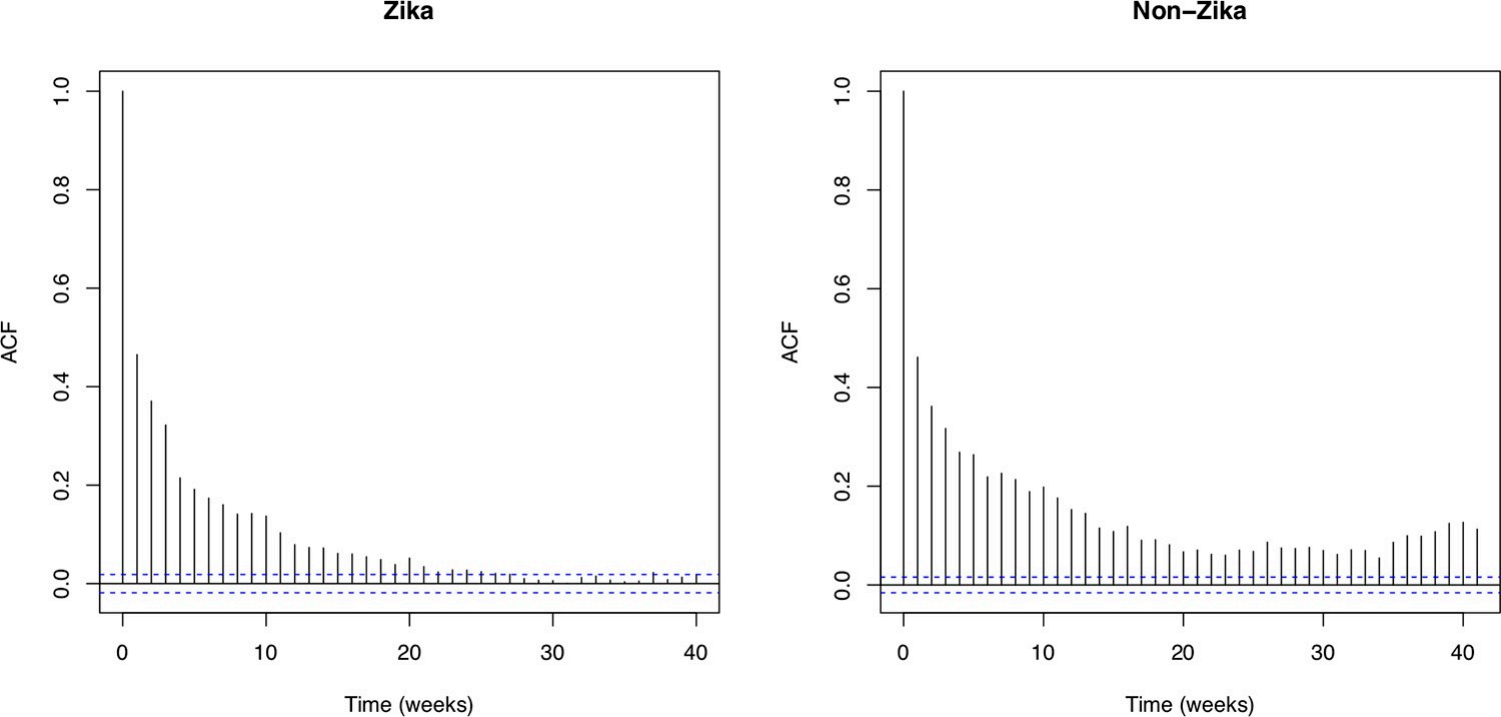
Temporal autocorrelation plot of average mosquito counts. These plots are shown over any one collection date for all traps in areas affected by Zika during the 2016 outbreak (left) and unaffected areas (right). Values outside of the blue-dashed lines are significantly different from zero.

*Ae*. *aegypti* populations fluctuate on both a weekly and a seasonal scale (Fig 2). The populations are highest in both regions in the summer months, peaking around July, and lower from mid-fall through mid-spring. *Ae*. *aegypti* populations in Zika regions are generally lower than populations in non-Zika regions over the summer months though the difference between the two is smaller in the winter months. Populations in both regions, especially during peak season, decreased slightly over the study period from 2016 to 2020. In Zika regions there is significant temporal autocorrelation between weekly-aggregated trap counts for up to 25 weeks, while for non-Zika regions there is significant temporal autocorrelation between weekly-aggregated trap counts for over 40 weeks (Fig 3). In both cases, this difference represents a period of time greater than the noticeable seasonal differences (Fig 2).

**Fig 4:**
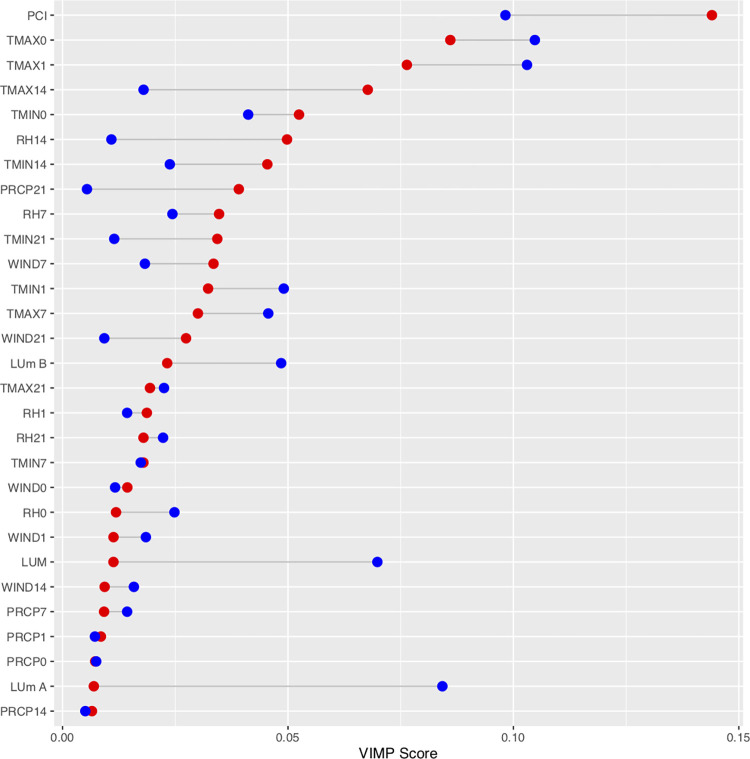
Variable importance (VIMP) scores for random forest runs. These scores are shown for models of *Ae*. *aegypti* adult females collected in traps in Zika affected regions of Miami (red) and areas of Miami unaffected by the Zika outbreak (blue). VIMP scores are “normalized” by modeling against the z-score of *Ae*. *aegypti* collected. Numbers next to variables indicate associated lag times.

Random forest model output for Zika and non-Zika regions indicates that the model explains about 40% of the variation in *Ae*. *aegypti* counts in the Zika region, 9% more than the model for the non-Zika region, and about 10% more than a model of all data across the county (Table 3). Variable importance scores show that per capita income and maximum daily temperature on the day of collection and the day before collection were the most important predictors for both models (Fig 4). For the Zika model this is followed by maximum daily temperature 14 days before collection, minimum daily temperature the day of collection, relative humidity fourteen days before collection, minimum daily temperature 14 days before collection, and precipitation 21 days before collection, whereas for the non-Zika model this is followed by major land-use and minor land-use A.

**Table 3:** Details of random forest models. Models were trained on data of adult female *Ae*. *aegypti* counts from traps in areas affected by local transmission during the 2016 Zika outbreak, traps in unaffected regions, and all traps.

	Zika	Non-Zika	All
**N (number of trap collections)**	11154	15213	26367
**Average number of terminal nodes**	1290.281	1876.577	3198.846
**Resample size used to grow trees**	7049	9615	16664
**Number of trees**	1000		
**Forest terminal node size**	5		
**Variables used**	Maximum temperature, minimum temperature, precipitation, relative humidity, wind speed (each at 0, 1, 7, 14, and 21 day lag times), major land-use, minor land-use A, minor land-use B, per capita income		
**R** ^**2**^	0.403	0.311	0.3042605

**Fig 5:**
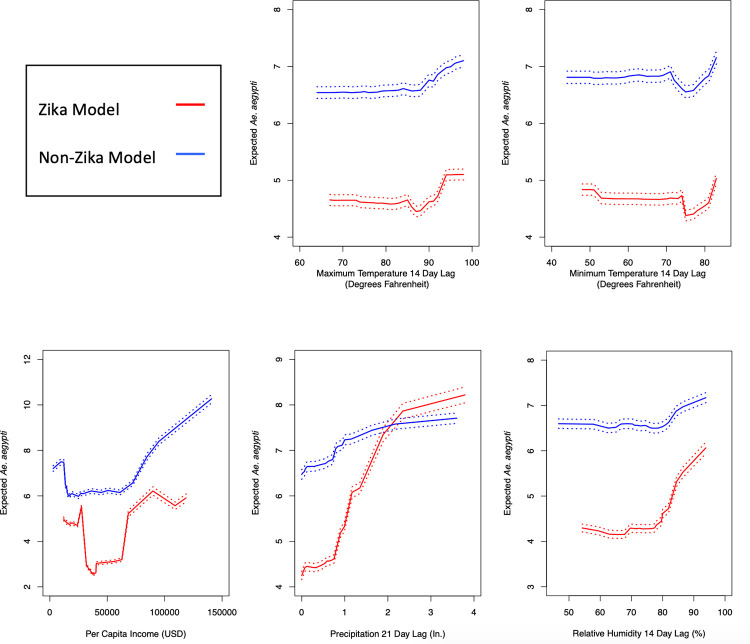
Partial plots of select variables in random forest models. These plots were generated for trap counts in areas affected by Zika during the 2016 outbreak (red) and unaffected areas. Dashed lines represent ±2 standard errors from expected *Ae*. *aegypti* counts per trap.

Partial plots of variables in the random forest model show the partial effect of each variable towards the model (Fig 5). Results indicate that maximum and minimum temperature at 14-day lag times contribute similarly to expected *Ae*. *aegypti* populations in the separate models for both the Zika and non-Zika region, with expected trap counts increase with temperature. For relative humidity 14 days before collection and precipitation 21 days before collection, an increase in either variable leads to a greater increase in the expected *Ae*. *aegypti* value in the models for the Zika regions than the non-Zika regions. The models also show that, for both regions, in low income areas there is a constant expected *Ae*. *aegypti* count, a dip in expected *Ae*. *aegypti* at medium incomes, and higher expected counts in higher income areas.

**Fig 6:**
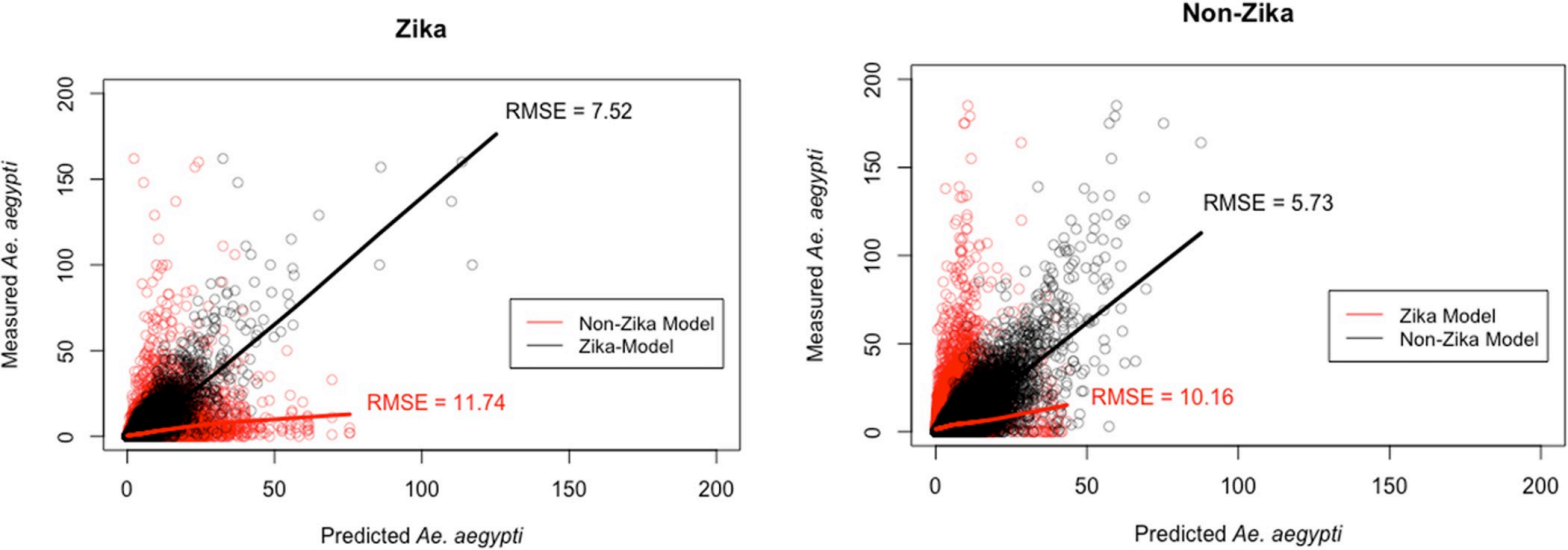
Predicted versus measured values for *Ae*. *aegypti* trap counts. These plots are shown for regions affected by local transmission of Zika during the 2016 outbreak (left) and unaffected regions (right), as predicted with random forest model trained on data from the respective region’s traps (black) or the opposite region’s traps (red).

By cross-predicting the data, differences in the models are shown (Fig 6). Attempting to predict the Zika counts with the non-Zika model results in a weaker prediction than with the Zika model (RMSE = 11.74 > RMSE = 7.52). Similarly, attempting to predict the non-Zika counts with the Zika model results in a weaker prediction than with the non-Zika model (RMSE = 10.16 > RMSE = 5.73). Differences in model predictions are further shown below (Table 4) using a set of predictors.

**Table 4:** Sample predictions for given model input conditions. Predictions were made using random forest models trained on data from traps located in regions affected by local transmission of Zika during the 2016 outbreak (“Zika model”) and unaffected regions (“Non-Zika model”). A full list of input parameters is included in the Methods section.

Scenario	Zika model expected *Ae*. *aegypti*	Non-Zika model expected *Ae*. *aegypti*
**Warm sunny summer day with rain three weeks prior in a medium-density medium-income residential area**	7.03	12.74
**Dry heat wave in high-density lower-income urban area**	7.51	12.20
**Colder rainy, windy day in medium-density medium-income residential area**	1.19	4.73
**Average conditions in low-income park**	2.37	2.04

## Discussion

The first hypothesis, that there is regional and temporal variation in *Ae*. *aegypti* populations throughout Miami-Dade County, is supported by this analysis. Temporally, while there is significant seasonal variation in the populations, the significant autocorrelation out to long-lengths, approximately half of a year or greater, in both regions implies that the populations do not rapidly fluctuate. However, we are unable to understand the variation in *Ae*. *aegypti* populations on a daily basis because of the nature of weekly collections. As weather conditions, especially precipitation, vary on a weekly basis, lacking daily data makes it hard to visualize the true temporal fluctuation in the data. More generally, it is possible that faster fluctuating vector populations may provide a greater risk for disease outbreaks, though this claim is not justified with prior studies. Spatially, there is significant variation in the number of *Ae*. *aegypti* collected per trap across different regions of the county. The traps with the highest average counts are only located in the more urbanized areas of the county (Fig 1). Even among nearby traps in these areas however, average trap count can vary greatly between traps in fairly close geographic proximity, indicating the major effects of the urban microgeography on *Ae*. *aegypti* populations.

The second hypothesis, that different regions of Miami-Dade County exhibit different relationships between weather variables and *Ae*. *aegpyti* populations, is also supported by this analysis. Random forest model results suggest that long-term weather variables are more important determining factors of mosquito populations in areas affected by Zika whereas day-of-collection weather conditions and land-use variables are more important determining factors of mosquito populations in unaffected areas (Fig 4). Minimum daily temperature, maximum daily temperature, relative humidity two weeks before collection, and precipitation three weeks before collection were all more effective predictors of trap counts in areas affected by Zika than in unaffected areas. Note from the partial plots (Fig 5) that not only were relative humidity and precipitation at longer lag times better predictors of *Ae*. *aegypti* trap counts, but they also had a larger effect on *Ae*. *aegypti* populations, as expected *Ae*. *aegypti* varied 50–100% over the relative humidity and precipitation lagged conditions in Zika regions and only about 20% in non-Zika regions. All three of the land-use values were much better predictors of *Ae*. *aegypti* populations in non-Zika regions than in Zika regions, suggesting that the local urban landscape is more important to determining these populations. Day-of-collection weather conditions may affect mosquito activity levels and trap strength, but they are not, in the majority of cases, predictive of or affecting the true vector mosquito population. Nonetheless, these conditions are more important determining factors in the number of mosquitoes trapped in non-Zika regions than Zika-regions.

The major result of this study is that *Ae*. *aegypti* populations across different microgeographies show varied responses to meteorological factors. Further, this variation may impact disease spread throughout an urban environment, as was seen with the spread of Zika in Miami-Dade County. This result is justified by and extends the current literature. Firstly, variations in Zika virus transmission on a fine spatial scale have been observed in studies in other areas [54, 55], which is evidence that similar microgeographic variations may be observed elsewhere. Potential mechanisms for why meteorological conditions may affect *Ae*. aegypti populations across urban micrographies differently include variable evapotranspiration or areas for water to collect, leading to differential habitat availability [56], variable environmental heat capacities, leading to different local temperatures [57, 58], and variable human density [59]. Another potential mechanism for why populations across different microgeographies may be affected differently by meteorological factors is through varied morphologies; populations in different urban environments have been shown to have different wing morphologies, potentially affecting their relationship with the meteorological conditions in their habitat [60]. The extension of this result is that faster-varying *Ae*. *aegypti* popluations, which are more strongly affected by weather conditions, may be more likely to spread the Zika virus or other vector-borne diseases. More prevalent virus spread in faster-varying mosquito populations may be due to population differences in virus transmission efficacy [61], different evolution rates in different microgeographies [62], and other unexplored factors.

There are several limitations to this study. Firstly, mosquito collections began in response to the Zika outbreak, so it is not possible to compare populations during the initial outbreak. As more traps were placed over the four-year period, trap quantity, spacing, and time frame were not constant between both regions. Simultaneously, mosquito control efforts around the county have not been controlled for in this study. Insecticide spraying, drainage of standing water, and other actions were taken by Miami-Dade County over the study period. These are factors that may contribute to the lower R^2^ values of the random forest models (Table 3). Additionally, they may contribute to populations in areas affected by Zika being generally lower than populations in unaffected areas as mosquito control efforts were focused more strongly on areas affected by local transmission of Zika [6] (Table 2). However, the random forest regression provides a method to understand the partial effects of relevant weather and spatial variables. Thus, while the overall models may be made weaker by greater variance in the data, the effects of the weather and spatial variables should be able to be inferred despite not accounting for population control efforts.

Other limitations of the study are a lack of trap-specific data on weather variables. All weather conditions may vary over the county. Weather conditions at each trap were not measured and therefore cannot be included in the analysis. This lack of data would only skew the results severely in the case that weather conditions vary differently over the Zika and non-Zika regions, but it is expected that both regions have similar variations in weather conditions. Temporal autocorrelation between weather variables at various lag times used is not too large for most variables but is necessary to mention for daily minimum and maximum temperature. For the entire prediction, the random forest model is not biased by collinearity between variables, as it will separate predictive strength between the variables in question. However, VIMP scores and partial plots may be affected for correlated variables as the model is forced to choose between variable importance and variable effect between similar variables. Despite the collinearity between different temperature lag times, the VIMP scores and partial plots of the temperature variables don’t appear to have been weakened, though further testing would need to be conducted to ensure no bias is added by the correlated temperature variables.

For Miami-Dade County Mosquito Control, these results provide evidence that the current approach to *Ae*. *aegypti* population control has been sufficient. Populations in each region have been gradually declining, especially during peak season, the time period most important for disease transmission. A regional approach to insecticide and larvicide spraying is justified when disease transmission is likely and *Ae*. *aegypti* populations are exceedingly high. The exact population thresholds will vary based on human populations and disease transmission efficiency and will need to be determined by individual modeling efforts. The individual result of this study, that rainfall at about three weeks lag time appears to be a more significant determining factor of mosquito populations in some regions than others, indicates that the “drain and cover” motto should target these regions especially. It should also target areas with lower average per capita incomes and residential areas. Drainage should occur as soon as possible after any large rainfall event (> 0.8 inches).

In future studies, it may be valuable to utilize more variables including vegetation at the trap site, human population density in the surrounding region, and populations of competing mosquito species which may all provide additional predictive strength. This analysis could be repeated in other cities on a neighborhood basis to understand which areas may be most susceptible to outbreaks from vector-borne diseases. More simply, it might also be mimicked with other disease-vector mosquito species in Miami-Dade County to observe if similar patterns hold, especially as the threat of other vector-borne diseases like Dengue virus and West Nile Virus remains.

These results show the complexity of mosquito populations in urban environments. Microgeographic conditions are determining factors throughout the entire life cycle of disease-vector species and dictate important spatiotemporal variance in their populations. In turn, spatiotemporal variability in these populations is the foundation for the spread of vector-borne diseases. These nuances, outlined here in this study and others, should guide the efforts of epidemiologists and vector control experts in modeling and reducing vector-species populations. As climate change continues to affect global weather patterns and as increasing urbanization changes habitats, vector-species populations are expected to shift their ranges and behaviors. As this change occurs, microgeographic analyses like those conducted here can play a critical role in protecting public health.

## Supporting information

S1 Table Variable values for sample predictions. Predictions are given in Table 4. Variable abbreviations can be found in Table 1. *Note that per capita income measured on university campuses are generally low as they capture student incomes.
(DOCX)
