# Supplementary material for: Using machine learning to understand microgeographic determinants of the Zika vector, Aedes aegypti
Source: PLoS One. 2022 Dec 30;17(12):e0265472. doi: 10.1371/journal.pone.0265472 (PMC9803113; doi:10.1371/journal.pone.0265472)
Supplement: S1 Table — Predictions are given in Table 4. Variable abbreviations can be found in Table 1. *Note that per capita income measured on university campuses are generally low as they capture student incomes. (DOCX) [file pone.0265472.s001.docx]

**S1 Table.** **Variable values for sample predictions**. Predictions are given in Table 4. Variable abbreviations can be found in Table 1. *Note that per capita income measured on university campuses are generally low as they capture student incomes.

| **Variable** | **Lag time (days)** | **Warm sunny summer day with rain three weeks prior in a medium-density medium-income residential area** | **Dry heat wave in high-density lower-income urban area** | **Colder rainy day in medium-density medium-income residential area** | **Average conditions at low-income park** | **Average conditions at university campus** |
| --- | --- | --- | --- | --- | --- | --- |
| **AWND (mph)** | **0** | 8 | 6 | 14 | 8 | 8 |
|  | **1** | 8 | 6 | 14 | 8 | 8 |
|  | **7** | 8 | 6 | 14 | 8 | 8 |
|  | **14** | 10 | 6 | 14 | 8 | 8 |
|  | **21** | 14 | 6 | 14 | 8 | 8 |
| **TMAX (degrees Celsius)** | **0** | 88 | 92 | 80 | 85 | 85 |
|  | **1** | 88 | 92 | 80 | 85 | 85 |
|  | **7** | 88 | 92 | 80 | 85 | 85 |
|  | **14** | 88 | 92 | 80 | 85 | 85 |
|  | **21** | 88 | 92 | 80 | 85 | 85 |
| **TMIN (degrees Celsius)** | **0** | 75 | 78 | 60 | 72 | 72 |
|  | **1** | 75 | 78 | 60 | 72 | 72 |
|  | **7** | 75 | 78 | 60 | 72 | 72 |
|  | **14** | 75 | 78 | 60 | 72 | 72 |
|  | **21** | 75 | 78 | 60 | 72 | 72 |
| **RH (%)** | **0** | 80 | 55 | 95 | 75 | 75 |
|  | **1** | 80 | 55 | 95 | 75 | 75 |
|  | **7** | 80 | 55 | 95 | 75 | 75 |
|  | **14** | 90 | 55 | 95 | 75 | 75 |
|  | **21** | 95 | 55 | 95 | 75 | 75 |
| **PRCP (In.)** | **0** | 0.2 | 0 | 2 | 0.2 | 0.2 |
|  | **1** | 0.2 | 0 | 2 | 0.2 | 0.2 |
|  | **7** | 0.2 | 0 | 2 | 0.2 | 0.2 |
|  | **14** | 1 | 0 | 2 | 0.2 | 0.2 |
|  | **21** | 2 | 0 | 2 | 0.2 | 0.2 |
| **LUM** | | MD | HD | MD | Park | Education |
| **LUmA** | | Road | Road | Road | Park | Road |
| **LUmB** | | Road | Road | Road | Park | MD |
| **PCI (USD)** | | 38,000 | 12,000 | 38,000 | 12,000 | 4,000* |
